# Asthma in the elderly: what we know and what we have yet to know

**DOI:** 10.1186/1939-4551-7-8

**Published:** 2014-05-30

**Authors:** Anahí Yáñez, Sang-Hoen Cho, Joan B Soriano, Lanny J Rosenwasser, Gustavo J Rodrigo, Klaus F Rabe, Stephen Peters, Akio Niimi, Dennis K Ledford, Rohit Katial, Leonardo M Fabbri, Juan C Celedón, Giorgio Walter Canonica, Paula Busse, Louis-Phillippe Boulet, Carlos E Baena-Cagnani, Qutayba Hamid, Claus Bachert, Ruby Pawankar, Stephen T Holgate

**Affiliations:** 1 Investigaciones en Alergia y Enfermedades Respiratorias- InAER, Buenos Aires, Argentina; 2 Department of Internal Medicine, Hanyang University Hospital, Seoul, South Korea; 3 Programa de Epidemiologia e Investigacion Clinica, Fundación Caubet-CIMERA, Illes Balears, Spain; 4 Children’s Mercy Hospital, University of Missouri – Kansas City School of Medicine, Kansas City, Missoui, United States of America; 5 Departamento de Emergencia, Hospital Central de las Fuerzas Armadas, Montevideo, Uruguay; 6 Krankenhaus Lungen Clinic, Grosshansdorf, Germany; 7 Wake Forest School of Medicine, Winston-Salem, North Carolina, United States of America; 8 Department of Medical Oncology and Immunology, Nagoya City University Graduate School of Medical Sciences, Kyoto, Japan; 9 Division of Allergy and Immunology, Department of Medicine, Morsani University of South Florida College of Medicine, James A Haley Veterans Hospital, Tampa, Florida, United States of America; 10 Division of Allergy and Immunology, National Jewish Health, Denver, Colorado, United States of America; 11 Department of Oncology, Haematology, and Respiratory Diseases, University of Modena and Reggio Emilia, Modena, Italy; 12 Division of Pulmonary Medicine, Allergy and Immunology, Children’s Hospital of UPMC, Pittsburgh, Pennsylvania, United States of America; 13 Respiratory Diseases and Allergy, University of Genoa, Genoa, Italy; 14 Division of Clinical Immunology, Department of Medicine, Mount Sinai School of Medicine, New York, New York, United States of America; 15 Institut universitaire de cardiologie et de pneumologie de Québec, (Quebec Heart and Lung Institute, Laval University), Quebéc, Canada; 16 Centre for Research in Respiratory Medicine, Catholic University of Córdoba, Córdoba, Argentina; 17 Meakins-Christie Laboratories, McGill University, Quebéc, Canada; 18 Upper Airways Research Laboratory (URL), Clinics ENT-Department, University Hospital Ghent, Ghent, Belgium; 19 Department of Pediatrics, Nippon Medical School, Tokyo, Japan; 20 Faculty of Medicine Clinical and Experimental Sciences, University of Southampton, Hampshire, United Kingdom

## Abstract

In the past, asthma was considered mainly as a childhood disease. However, asthma is an important cause of morbidity and mortality in the elderly nowadays. In addition, the burden of asthma is more significant in the elderly than in their younger counterparts, particularly with regard to mortality, hospitalization, medical costs or health-related quality of life. Nevertheless, asthma in the elderly is still been underdiagnosed and undertreated. Therefore, it is an imperative task to recognize our current challenges and to set future directions. This project aims to review the current literature and identify unmet needs in the fields of research and practice for asthma in the elderly. This will enable us to find new research directions, propose new therapeutic strategies, and ultimately improve outcomes for elderly people with asthma. There are data to suggest that asthma in older adults is phenotypically different from young patients, with potential impact on the diagnosis, assessment and management in this population. The diagnosis of AIE in older populations relies on the same clinical findings and diagnostic tests used in younger populations, but the interpretation of the clinical data is more difficult. The challenge today is to encourage new research in AIE but to use the existing knowledge we have to make the diagnosis of AIE, educate the patient, develop a therapeutic approach to control the disease, and ultimately provide a better quality of life to our elderly patients.

## Introduction

We are in an unprecedented era of rapid aging of the global population. Demographic projections estimate that the number of elderly people will double in many regions by 2030 [1]. In the past, asthma was considered mainly as a childhood disease; however, recent epidemiologic studies have indicated that asthma is highly frequent in the elderly population with its prevalence ranging from 4.5% to 12.7% [2-15]. In addition, the burden of asthma is more significant in the elderly than in their younger counterparts, particularly with regard to mortality, hospitalization, medical costs or health-related quality of life [15-20]. Nevertheless, asthma in the elderly (AIE) is still been underdiagnosed and undertreated [5,21-23].

Notably, AIE may be considered a late-onset disease [14-23]. The French elderly population cohort 3C study, reported that asthma incidence among the elderly was 3.0/1,000 person-year [14]. In Italian general population surveys, the asthma incidence rate after 40 yrs old was 2.27/1,000 person-year, which was increasingly higher with aging from the third decade of life [24]. Along with this, AIE might have a different pathophysiology than in childhood disease, resulting from complex interactions with various factors such as aging-related lung and immune alterations, epigenetic factors, environmental exposures, microbial triggers, or various comorbidities [19].

However, our understanding of this ‘old but new’ disease is still not complete. Prior knowledge was mostly based on experimental or clinical studies targeted for allergic or Th2-mediated asthma, which is not a predominant feature in AIE [19]. Clinical studies for asthma treatment have often excluded elderly subjects [23]. Even epidemiologic observational studies have been scarce, which would provide observational findings for understanding its nature or pathophysiology.

Therefore, it is an imperative task to recognize our current challenges and to set future directions. The primary aim of the present World Allergy Organization project is to review the current literature and identify unmet needs in the fields of research and practice for AIE. This will enable us to find new research directions, propose new therapeutic strategies, and ultimately improve outcomes for elderly people with asthma.

## Life expectations, lifespan and maximum survival

From the perspective of studying aging, there is a significant difference between average and maximum life span. The average life span is the average age reached by members of a given population, and life expectancy is the number of years an individual can expect to live. On the other hand, the maximum life span refers to a measure of the maximum amount of time one or more members of a population has been observed to survive between birth and death [19].

Over the past decades, with the introduction of modern sanitation, refrigeration and other public health measures including vaccination, antibiotics, and aggressive cardiovascular preventive as well as surgical procedures, there has been an increase in average life span [25]. Early deaths have been diminished and more individuals are reaching old age. In the United States today, life expectancy now approaches 80 years [26]. However, the maximum life span, which is 122 years old, has remained unchanged by the public health initiatives mentioned above [19].

Average life span is what concerns public health officials and health care providers but for those studying the biology of aging, it is maximum survival that is the focus of greatest attention. This maximum is believed to provide a more meaningful indicator of the underlying rate of aging because the average life span may be prolonged entirely because of an optimization of the maintenance conditions rather than a slowdown of the rate of aging. It is worthwhile to note that it has been estimated that if atherosclerosis and cancer were eliminated from the population as a cause of death, about ten years would be added to the average life span, yet there would be no change in maximum life span [27].

Although several theories have been proposed, none suffice to account for the complexities of aging. Life span is finite and varies generally from species to species and much less so within species. Variations in maximum life span among different species are often associated with differences in the metabolic rates of oxygen consumption, metabolic potential (estimated as the total amount of energy consumed per gram of body weight during the life-span), and the level of oxidative stress [19].

A causal mechanism of aging, implicating the endogenously generated oxygen free radicals as the agents of damage, was first proposed by Harman in 1956 [28]. Although experimental augmentation of antioxidant defenses tends to enhance resistance to induced oxidative stress, such manipulations are generally ineffective in the extension of life [29]. More recently, in a major conceptual shift, reactive oxygen species have been found to be physiologically vital for signal transduction, gene regulation, and redox regulation, among others, implying that their complete elimination would be harmful. An alternative notion, termed the "redox stress hypothesis," proposes that aging-associated functional losses are primarily caused by a progressive pro-oxidizing shift in the redox state of the cells, which leads to the over-oxidation of redox-sensitive protein thiols and the consequent disruption of the redox-regulated signaling mechanisms [29].

Three regimes are known to extend the maximum life span of animals: (i) lowered ambient temperature in poikilotherms (cold-blooded animals) and hibernating mammals and (ii) a decrease in physical activity in poikilotherms, both of which decrease metabolic rate, and (iii) caloric restriction [19].

Caloric restriction is now being increasingly used as a model regimen for understanding the basic mechanisms of aging, primarily because it causes an unambiguous, robust, and reproducible extension of maximum life span and delays many, although not all, age-associated biochemical, physiological, and behavioral changes [19].

The extension of maximum life span by caloric restriction in mammals and hypometabolic states in poikilotherms, point toward the involvement of environmental-genetic interactions in the process of longevity. However, the existence of specific gene products, that initiate deleterious alterations in the latter part of life, has not as yet been demonstrated [29]. As stated by Hayflick [30], genes do not directly drive the aging process, rather they indirectly modulate the potential life span by specifying a certain level of physiological fitness, determined by the efficiency of functions such as repair, turnover and replacement. Accordingly, the progression of senescent deterioration can be envisioned to be mainly dependent upon the genome-controlled efficiency of the physiological systems to maintain homeostasis, and the magnitude of the stochastic events that diminish the ability of the organism to maintain homeostasis. Thus, the pathways/mechanisms involved in resistance to various types of stress and maintenance of bioenergetic capacity and redox homeostasis may be critical in the evolution of longevity [29].

**Figure 1:**
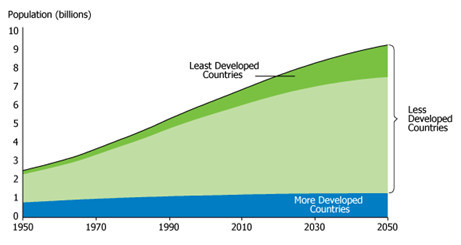
World Population Growth trends from 1950 and projected to 2050.

As of today, there have never been more people, more elders, and more smokers than ever before in the history of mankind. The World Health Organization estimates the ever increasing World population grew to 7.06 billion in mid-2012 after having passed the 7 billion mark in 2011 (Figure 1). Most of this growth is occurring in developing countries [31].

**Figure 2:**
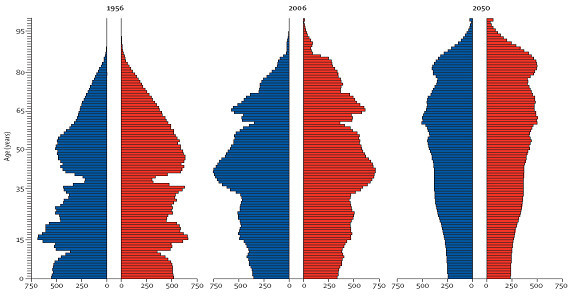
World Population age-and gender-structure in 1956, 2006 and projected to 2050.

Further, with 1.1 billion there have never been as many smokers as of today [31]. Finally, the world population is ageing, with an inverted age- and gender-pyramid structure soaring (Figure 2), and it is considered that most babies born since 2000 in many countries (i.e.: France, Germany, Italy, the UK, the USA, Canada, Japan, and other countries with long life expectancies) will celebrate their 100th birthdays [32].

Hence, the numbers of older people with cancer, fractured hips, strokes, and dementia will increase, and many older people will have multimorbidities. One might think that this will bring a formidable challenge to many countries. However, projected increases in health expenditure as a result of ageing are slight and ageing does not present a fundamental threat [33].

Asthma is considered a common condition (the most frequent in children), mostly with mild symptoms at the population level, and with relatively good individual prognosis [32].

Individuals with asthma are/will be living the same life expectancy as those with no asthma, therefore will require asthma treatment and monitoring for decades. In the latest Global Burden of Disease update, asthma ranked 14^th^ in the classification of Years Lived with Disability, and totaled counts of 334 million pople with asthma worldwide [34].

## Impact of AIE

Asthma is a common disease affecting individuals across the lifespan. Because of increased longevity, the proportion of individuals aged 65 years and older (heretofore referred to as the elderly) is increasing worldwide. By 2030, elderly subjects will comprise ~20% and ~36% of the populations of the United States (U.S.) and China, respectively [35,36]. Given these demographic changes, the fact that asthma is already common in elderly subjects (see below), and the inevitable aging of children affected by the “asthma epidemic” in the second half of the 20^th^ century [37], the impact of AIE is expected to be magnified over the upcoming decades.

Determining the exact prevalence of AIE is made difficult by barriers including: 1) under-diagnosis due to decreased perception or under-reporting of symptoms by patients [21], 2) suboptimal utilization of spirometry and other diagnostic tests, 3) misclassification of asthma as chronic obstructive pulmonary disease (COPD) and *vice versa*, 4) failure to recognize asthma in subjects with co-morbidities such as congestive heart failure or COPD, and 5) exclusion of elderly subjects from population-based studies of asthma. In spite of these challenges, current evidence consistently suggests that asthma is common among elderly subjects.

In two nationwide surveys in the U.S. (conducted using similar methods), estimates of the prevalence of current asthma (defined as physician-diagnosed asthma [ever] in subjects with active disease) in the elderly were 3.6% for the period comprising 1988 to 1994 [38] and 5.9% for the period comprising 1980–2004 [39]. In a community-based cohort study of elderly subjects in the U.S., “definite asthma” (defined in the same manner as “current asthma” in the two studies above) and “probable asthma” (defined as wheezing accompanied by chest tightness or shortness of breath in the previous year) were each estimated to be present in 4% of non-smoking elderly participants without congestive heart failure [21,38-41]. In this study, estimates of the prevalence of current and “probable” asthma in all subjects were 11% and 14%, respectively. In elderly subjects, asthma is more common in women than in men [39]. The 5-year age- and sex-specific incidence of new cases of asthma in subjects older than 65 years has been estimated to be approximately 1 in 1,000 [40].

Compared to children or younger adults, older adults and/or elderly subjects have greater morbidity and healthcare costs from asthma. In 2012, Tsai *et al.* published the results of an analysis of a large nationwide U.S. database for emergency department (ED) visits and hospitalizations between 2006 and 2008 [42]. Among subjects who visited the ED for asthma, those aged 55 years and older had higher rates of hospitalization and near-fatal asthma events, higher hospital charges, longer hospital length of stay and higher overall mortality than adults aged 18 to 54.9 years (P <0.001 in all cases). Similar findings were obtained after adjusting for co-morbidities and other covariates, or after excluding subjects having COPD-related secondary diagnoses [41]. Charges for ED visits, hospitalizations, prescription medications and management of co-morbidities predictably result in substantial cost burdens attributable to AIE [18,42].

Older adults (including the elderly) have been shown to have a higher overall (all-cause) mortality than younger subjects with asthma in most [42-44,46-48] but not all [45] published studies. From 2001 to 2003, the estimated rates of all-cause mortality among subjects with current asthma in the U.S. were substantially higher in elderly subjects (~10.5 per 100,000) than in subjects in other age groups (<2.2 per 100,000 in all cases)^6^. In the recent study by Tsai *et al.*[42], elderly subjects (ages 65 years and older) had fourfold greater overall mortality than subjects ages 18 to 64.9 years (odds ratio = 4.1, 95% confidence interval = 3.1 to 5.5).

## The aging lung: the role of the inflammatory, allergic and infectious process

### Structural, cellular and physiologic changes in the aging lung

#### ***Respiratory mechanics***

Respiratory mechanics involves an interplay between the chest wall, lung and diaphragm. Because of simultaneous changes in the mechanical properties of the lungs and chest wall and the interdependence of muscle groups, it is difficult to precisely quantify age-related changes of specific respiratory muscles [49]. Respiratory muscle strength, particularly that of the diaphragm, can be assessed by measuring the maximum voluntary ventilation (MVV) and maximum inspiratory pressure (MIP). MVV is reduced with age [50], and the clinical risks associated with such changes include diaphragmatic fatigue and potential ventilatory failure during increased ventilatory load. MIP is a measure of diaphragmatic strength and has also been shown to decrease between the ages of 65 and 85 years [51,52].

Given the similarities between the aging lung and the lung affected by COPD, some speculate that COPD actually represents an accelerated lung aging phenotype [53]. The aging lung mimics emphysema in that both have enlarged alveoli, decreased surface area for gas exchange, and decreased elastic recoil, leading to the term “senile emphysema” that has been used to describe the normal aging lung. This term is not fully accurate, however, since the normal aging lung lacks airway wall destruction and distal duct ectasia classically seen in emphysema, and the changes of normal aging are more homogeneous than those of emphysema [54-56]. What is not clear is whether “senile emphysema” reflects aging-associated destruction of lung parenchyma or loss of supporting structures [57]. There is degeneration of the elastic fibers around the alveolar duct starting around 50 years of age, resulting in enlargement of airspaces. Dynamic CT imaging has shown emphysematous change with age [58]. However, a definitive progression to an emphysema phenotype will never be proven because this would require serial biopsies over many years.

#### ***Cellular***

Ultimately, the major effect of aging on the immune system may be a shift from naïve to memory lymphocytes and an increased presence of the innate immune system, contributing to low-grade chronic inflammation [59-61]. Although the effects of aging on adaptive immunity have been studied more extensively (they will be described later in this chapter), several features of innate immunity may also change with aging. In animal studies, macrophages have been noted to decrease their expression of toll-like receptors (TLRs), their secretion of cytokines following activation, and their phagocytic ability. This decline occurs in parallel with decreasing levels of macrophage-derived chemokines [59,60]. The effect of aging on monocyte and macrophage function in humans, however, remains controversial. Although some studies suggest that aging does not affect the function of these cells, others have demonstrated an impaired cytotoxic effect of aged monocytes [62]. In addition to impaired function, monocyte/macrophage and lymphocyte recruitment to infected tissue has been shown to be delayed in elderly individuals based on a study of cutaneous punch biopsies [63].

Examination of cell types and molecules in the BAL fluid of older adults supports the theory of low-grade chronic inflammation in the aging lung. It has been demonstrated a higher percentage of neutrophils and a lower percentage of macrophages [64]. This increased proportion of neutrophils in BAL fluid with age was also noted in two subsequent studies by Meyer *et al.*[65,66]. A significant increase in total cell concentration, neutrophils, immunoglobulin content, and IL-6 concentration was observed in the BAL fluid from older adults [65,66]. BAL samples from the older individuals demonstrated more IL-8, neutrophil elastase, and several antiproteases, consistent with an increased neutrophil predominance in these older individuals [67]. These observations suggest that even asymptomatic and clinically normal older volunteers have altered inflammatory profiles, reflecting the presence of low-grade inflammation in the lower respiratory tracts. These alterations include a significant rise in the number of CD4+ T cells, neutrophils, immunoglobulins, and cytokines such as IL-6 and IL-8, as well as an increase in the release of superoxide anion and other byproducts of neutrophil activation [65-67].

#### ***Physiologic changes***

Even in the absence of disease, there is a predictable loss of lung volume with advancing age. The lungs undergo growth and maturation approximately to age 20, at which point maximal function is achieved. Lung function remains static through the third decade and into the fourth decade of life, after which a decline in pulmonary function begins. Most cross-sectional studies show a linear decline in FEV1 with age, whereas longitudinal studies show a nonlinear decline with age. The estimated rate of decline in FEV1 is initially 25–30 ml/yr. starting at age 35–40 and can double to 60 ml/yr. after age 70 [68]. However, the actual rate of decline is difficult to assess as inter-individual variability exceeds the decline reported based on predictive modeling.

The ratio of FEV1 to FVC is lower in older healthy people; therefore, the use of a fixed cut like 0.70 or 80% as the lower limit of normal will result in the over-diagnosis of obstructive airway disease in older adults [69-73]. Data collected from the Cardiovascular Health Study have suggested that the lower limit of normal for FEV1/FVC should be 64% to 56% for persons aged 65 to 85 years, respectively [74]. Finally, the clinical implications of such age-related decline must be factored in with the maximal attainable lung function.

### Age related changes in adaptive immunity

#### ***Changes of lymphocyte function with age***

Lymphocytes clearly change with aging. There is a reduction primarily in thymic function as T cells age, and the number of naïve lymphocytes in the thymic compartment as well as in the periphery is significantly reduced with aging. As a direct consequence, the actual mass of T cells and B cells probably undergoes at 25% reduction with aging. The causes of this age associated decline clearly relate to the decline in the generation of naïve cells [75,76]. Nave CD4 cells isolated from older humans have decreased in vitro responsiveness to activation through T cell receptor pathways, but also through different cytokine usage and secretion patterns when compared to naïve CD4 cells taken from young individuals. CD4+ T cells and loss of function of signaling proteins with aging may reduce the efficiency of this synaptic activation between antigen presenting cells and T cell receptors [76,77]. Memory T cells which are long lived and maintained by homeostatic cytokines remain relatively competent even into old age, but the numbers of these memory CD4 T cells are reduced [77]. The differences in B cell activation as aging occurs may play a potential role in the increase in significance of autoimmunity and selection on B cell clones that are not common clones that can lead to altered autoimmune immunity at the B cell level. B cell related aging also uncovers a lack of normal responses. It is also possible that memory B cells that may be maintained that are of little value in terms of maintaining a protective vaccination status with reduced T cell activation [78].

#### ***The mechanisms of aged lymphocyte dysfunction***

Altered expression of chemokines and cytokines via actual production as well as receptor expression are found in both human T cells and murine models of aging. Immunosenescence and clonal energy is also a significant contributor to reduction T cell activity in aging individuals. These senescent T cells escape apoptosis but are unable to be activated with this normal and standard means of activation and may identify both T and B cell compartments of clonal cells that are non responsive and are of very little use in the terms of host defense and/or maintenance of vigilance against disease [77,78].

#### ***Implications for disease and physiological responses***

The fact that aging T cells fail and have reduced function is of concern for three reasons. The first is inadequate responses to vaccination as elderly individuals are vaccinated receiving pneumovax and other anti pneumonia agents, some directed at bacteria and some directed at viruses. The development of an actual significant immune response is critical in the CD4, CD8 and B cell compartments, but the reduction in size and function of these compartments correlates with difficulty in achieving proper vaccination and it should be examined in any elderly individual receiving a vaccination. Hence, the efficacy of the vaccination process needs to be tested in those individuals and the revaccination and booster activities should be put in place of those who make a less than adequate response to any vaccine. In addition, the lack of vigilance related to the T cell compartment through help of NK cells and CD8 T cells is also of consideration and potential development of both autoimmune diseases and also hypersensitivity diseases including asthma for quite possibly other lung disease including susceptibility to ARDS with infection and susceptibility to other viral lung infections [78].

### Allergen sensitization in older adults

The role of atopy and asthma in older subjects, unlike in children and young adults, is not completely understood. Over 80% of young children with asthma are allergen sensitized, and atopy in this age group increases disease morbidity [79,80]. Additionally, atopy plays a critical role in the inception of asthma in this age group, in particular during viral infection [23]. However, with increased recognition of asthma in older subjects, our knowledge on the role of IgE sensitization to antigens and subsequent exposure to subjects with asthma in this age group is increasing. It is well established that with increased age, *total* serum IgE decreases. This has been demonstrated in several cross-sectional studies of randomly selected individuals. For example, the Tucson Epidemiological Study and the National Health and Nutrition Examination Survey (NHANES 2005–2006) reported that IgE peaks by 20 years of age and is lowest after 70 years [81,82]. However, not all studies have not supported this trend. [83,84]. Additionally, when looking at the prevalence of allergen-specific IgE, younger populations, including subjects with and without asthma, tend to have a higher prevalence of allergen-specific IgE sensitization than in older groups [83,85]. This has been reported in several longitudinal studies from cohorts of randomly selected subjects from Tucson [85], Nottingham [86], Copenhagen [87], and in the European Community Respiratory Health Survey [88].

Although it is generally accepted that antigen-specific IgE sensitization decreases with age, the prevalence of older patients with asthma who are atopic is not clearly established. For many years, asthma in older patients was characterized as non-atopic or intrinsic [89]. Over the past two decades, there have been a few reports investigating older patients with asthma, which have demonstrated that atopy (defined as IgE-sensitization to at least one antigen) is not uncommon in this group. The reported percentage of older patients with atopic versus non-atopic asthma may depend upon the characteristics of the population studied. Studies done in non inner- city populations are variable; suggesting that 28% to 74% of older adults with asthma are sensitized to at least one antigen [90-93]. However, there aappears to be a higher rate of allergen sensitization in older patients with asthma compared to age-matched controls without asthma, suggesting a difference with asthma [90,92].

Two studies have investigated antigen sensitization rates to common aeroallergens in older asthmatics from US inner-city populations. Rogers *et al.* reported that in an asthma clinic in New York City , that 60% of subjects > 65 years of age had at least one detectable allergen specific IgE (including outdoor allergens) and that cockroach sensitization was the most prevalent at 47% [94]. Cockroach sensitization was associated with more severe asthma as determined by airflow limitation and hyperinflation. This study did include a group of younger subjects from the same population for comparison. Another study done in New York City reported that 41% of subjects over the age of 60 years with moderate to severe persistent asthma, were sensitized to at least one antigen, whereas 73% between 18–35 years of age were sensitized [95]. Antigen sensitization developing later in life may contribute to late onset asthma in some patients [96,97]. In the Normative Aging Study, which followed subjects over an extended period of time, those men who developed airway hyper-responsiveness after 49 years of age were more likely to have developed recent IgE to cat (23.9% versus 4.4%) compared to age-matched controls [96]. Additionally, approximately 50% of the forty patients in the Tucson Epidemiologic study of obstructive lung diseases who developed asthma after the age of 60 years were skin prick positive to at least one antigen, compared with 26% of the age-matched control population without asthma [90]. In a study of 21 patients with asthma onset after 65 years of age, 81% demonstrated a positive skin prick test to at least one allergen compared to a group of 14 patients developing asthma before the age of 65 years in whom 57% were allergen sensitized [96]. A French study recruited 1,485 patients (mean age 73 years) with a diagnosis of asthma to examine disease characteristics. 14.7% of those developing asthma after 65 years of age were sensitized to at least one antigen by skin prick testing, whereas 60.1% of those developing asthma prior to 21 years of age were antigen sensitized [91].

The most common aeroallergen to which older patients with asthma are sensitized is not consistent among reports, but includes cat [93], dust mites [92,95], and cockroach [94]. Whether the differences in specific antigen sensitization are due to socioeconomic status, geographic location and environmental exposures, is not well established at the present. An important unanswered question is, what is the role of allergen exposure in sensitized older patients with asthma on disease pathogenesis and severity?

There is emerging evidence for Staphylococcal enterotoxin (SE) sensitization as a major risk factor for adult asthma [98-100]. Particularly, it is suggested to have relationships with intrinsic asthma [101], or severe non-atopic late-onset asthma [102]. In recent GA2LEN surveys, the prevalence of SE IgE sensitization was 29.3% in the European adult populations, which was higher than that of house dust mite (14.9%) [100]. Of note, they found that the SE sensitization was positively related to smoking history and aging. As Staphylococcus aureus is a frequent colonizer in the upper airways and skins, the airway epithelial disruption by repeated smoke exposure [103] or the reduced cutaneous barrier function by aging process [104] could contribute to the SE IgE sensitization. Thus, ‘aging’ could be a predisposing factor for SE sensitization and also for developing asthma in later adult life among susceptible subjects. Although there is still no direct evidence, the collective evidence suggests the potential contributions of SE sensitization on the nature of elderly asthma. The atopic condition in the geriatric age group represents an additional tool in the diagnostic process and, consequently, in the therapeutic approach [105].

### Immunosenescence and infection

Immunosenescence affects both the innate and adaptive immunity [106,107]. Major clinical impact of immunosenescence is an increased susceptibility to microbes, such as viral or bacterial infection. The health care cost is larger for older people than young people as a result, at least in part, of the increased susceptibility to infectious diseases and reduced immune responses to vaccination with aging. For example, older subjects exhibit a higher mortality rate to influenza viral infection compared to younger subjects [108]. Therefore, it is important to understand the impact of aging on the immune system.

In older asthmatics as well as in children, viral respiratory infection is associated with worsening of asthma control [109]. In a prospective cohort study among healthy elderly subjects and high-risk subjects (those with chronic heart or lung disease), respiratory syncytial virus infection was observed annually in 3 to 7% of healthy subjects and in 4 to 10% of high-risk subjects. On the basis to the diagnosis at discharge, RSV infection accounted for 10.6 percent of hospitalizations for pneumonia, 11.4 percent for chronic obstructive pulmonary disease, 5.4 percent for congestive heart failure, and 7.2 percent for asthma [110].

Vaccination is an effective approach to sustain immune responses and prevent the deterioration of infectious disease for elderly subjects. In general, commonly used vaccinations against influenza virus and *pneumococcal* pneumonias are effective at preventing the development of these infectious diseases among the elderly subject [111,112]. Among asthmatics, vaccination against influenza virus has been shown to help prevent asthma exacerbations in children but there is less [113], but less evidence is present in elderly asthmatics. Pneumococcal vaccination is recommended in COPD patients, but its value in adult asthmatic patients is less certain [114].

In summary, the immune system declines with age, and elderly asthma patients are more prone to airway infection than younger subjects. However, studies exploring the association between asthma and infection have mainly targeted pediatric patients. In the future, clinical and experimental studies focusing on elderly subjects are expected to clarify the role of immunosenescence in the pathophysiology of asthma.

### Role of upper airway diseases in elderly asthma

Relationships between asthma and upper airway diseases have been consistently observed across various age groups [115-117]. However, the mechanisms of their associations may be multifactorial, including atopy, microaspiration, nasopharyngo-bronchial reflex, or systemic pathway [118]. In the past, the role of inhalant allergen sensitization has been considered as a major factor to predispose the development of asthma in subjects with allergic rhinitis [119]. However, rhinitis and asthma in the elderly appear to be mostly non-atopic but still show significant associations [120], suggesting the presence of further mechanisms in the aged population.

With regard to this, recent evidence indicates the potential roles of chronic rhinosinusitis (CRS) in the asthma pathogenesis. The GA2LEN surveys found that late-onset adult asthma was independently associated with CRS irrespective of nasal allergies [121]. Recent endotype approaches suggest the specific roles of Staphylococcal enterotoxin sensitization in the pathogenesis of CRS subtypes with nasal polyp [122] and severe late-onset non-atopic adult asthma [102]. These findings may also be quite relevant to the elderly population, as elderly asthma is a considerably late-onset disease.

## Diagnosis and clinical assessment: with special emphasis on the clinical features of the overlap between COPD and asthma

Asthma is underdiagnosed in the elderly due to misattribution of symptoms and signs to other diseases common in the aged, such as COPD or heart disease, or acceptance of symptoms and limitations as a normal result of aging. Thus, the clinician must remain more vigilant to recognize asthma in older patients [123].

Aging influences the symptoms of asthma and the risk of mortality. This may be due to changes in airway physiology with aging and the decreased response to treatment [124,125].

### Physiology and making the diagnosis of asthma

Lung function decreases with age due to increased stiffness of the chest wall, reduced respiratory muscle function and an increase in residual volume from loss of elastic recoil. The decline in the elasticity of the airway is considered the major contributor to the increase in fixed airflow obstruction and work of breathing with age. The result is a decrease in FEV1/FVC, such that normal elders have spirometric features suggestive of obstructive lung disease. Thus, the diagnosis of AIE is challenging, and AIE is commonly misdiagnosed as COPD resulting in the under-diagnosis and under-treatment of asthma [126]. Significant, irreversible airflow obstruction in older adults is usually due to COPD, while remodeling or bronchiectasis with segmental fibrosis is more characteristic of older adults with asthma. Patients with COPD often have increased lung volume (air trapping), reduced diffusion capacity, and emphysematous changes on high resolution tomographic imaging (often absent on chest radiographs). When these findings are present, patients with persistent dyspnea and reduced FEV1 of less than 60% predicted are more likely to have COPD than AIE [127].

It has been suggested that, overall, the prevalence of airway hyperresponsiveness increases with age, and there is a positive correlation between age and airway hyperresponsiveness recruited subjects aged > 65 yrs [128,129].

The clinical significance of these observations lies in the fact that the elderly population is particularly at risk of developing persistent airway closure. The important question is, therefore, whether measurements of airway hyperresponsiveness are valuable in elderly individuals, in whom the perception of symptoms may be blunted. Scichilone *et al.* propose that assessment of airway hyperresponsivenes in the elderly should be considered an additional tool in the diagnostic work-up of subjects who belong to the at risk group [128].

AIE may also have specific phenotypes. It appears that age of onset, and thus the duration of asthma, may be important in delineating at least two such phenotypes: late-onset asthma (LOA) with onset after middle age, and long-standing asthma (LSA) with onset in childhood or in early adulthood. Although atopy is commonly associated with both phenotypes, allergies and obesity are commonly associated with LSA, but much less likely to be associated with LOA [126,127].

### Clinical features

The symptoms of asthma and COPD are very similar, if not identical. The major differences are the degree of reversibility is often greater in asthma and the persistence of dyspnea is greater in COPD. Both are characterized by exacerbations which respond to corticosteroids and bronchodilators, infections triggering exacerbations, episodic wheezing, cough with or without mucous production, improvement with chronic inhaled corticosteroids and bronchodilators, and decreased exercise tolerance. The chronic bronchitis phenotype of COPD as opposed to the emphysematous phenotype is more likely to be confused with asthma, but the variations of phenotypes in both diseases confounds simple measures to reliably distinguish one from another [126,127]. The major distinguishing clinical features between the two diseases are personal or family history of atopy and/or asthma with symptoms starting in childhood being more likely with asthma; cigarette smoking history and adult onset of symptoms being more likely in COPD; and increased biomarkers including fractional exhaled nitric oxide, peripheral and sputum eosinophil and serum total and specific IgE being more likely with asthma [95]. However, exceptions are not unusual, and the LOA phenotype may not exhibit many of the distinguishing factors for asthma [127].

### Overlap between COPD, asthma and other diseases with fixed airflow obstruction

In addition to the diagnostic challenges resulting from spirometric changes of aging and other disease processes that share similar clinical presentations, the identification of triggers for AIE are more difficult to define. Infections are the most important triggering factors, similar to COPD [126,127]. Compared to the younger asthmatic, the role of specific IgE in AIE is decreased, and a positive family history of asthma is less common, particularly in the LOA phenotype [95]. In addition, due to changes in the skin from normal aging and damage from sun exposure in the elderly, the responses of skin testing are smaller in induration, have less erythema and are less consistent. Thus, the interpretation of both allergen skin tests and in vitro specific IgE testing is confounded in the elderly [95,130]. Peripheral blood or airway eosinophilia, characteristic of asthma is useful in distinguishing COPD from asthma. Also the severity of upper airway disease is likely to be more significant in asthma compared to COPD [126,127].

Cough is an important symptom of asthma but in the elderly the source of cough is often multifactorial. The other conditions or disorders causing cough in older adults include laryngopharyngeal reflux, pulmonary congestion from heart disease, COPD, angiotensin converting enzyme inhibitor therapy, airway dryness from Sjögren syndrome or the drying effects of other medications, aspiration due to swallowing dysfunction, pulmonary fibrosis, bronchiolitis or bronchiectasis. Chest imaging and review of medication lists may be very helpful in distinguishing these other causes of cough from the cough from asthma.

A restrictive component of decreased airflow would suggest pulmonary fibrosis, bronchiectasis, chest wall restriction from prior surgery or injury, calcification of costal cartilage or scarring of the lung from prior infection, or chronic aspiration. Finally, the coexistence of more than one disease in older adults is more likely, further challenging diagnostic certainty [127]. Given that the prevalence of smoking in asthmatics mimics the prevalence of smoking in the general population in that country [131], all asthmatics should have their smoking status assessed, and offered individualized anti-smoking strategies, both to improve their asthma and overall health.

### Upper airway comorbidities

Whereas younger patients with asthma often suffer from allergic rhinitis complaints, elderly patients with asthma often suffer from sinus symptoms, including nasal obstruction, loss of smell and facial pain/headache. A recent Europe-wide epidemiologic study on the prevalence of chronic rhinosinusitis (CRS) did confirm the well-known association between allergic rhinitis and early-onset asthma, but also demonstrated a clear increased risk to suffer from late-onset asthma in CRS patients [132]. CRS may be phenotyped as CRS without (CRSsNP) and with nasal polyps (CRSwNP), based on symptoms (loss of smell is typical for CRSwNP, headache and facial pain are typical for CRSsNP), nasal endoscopy (presence of bilateral nasal polyps) and CT scanning. From those phenotypes, CRSwNP has a clearly increased risk of asthma comorbidity in Caucasian populations [133], whereas CRSsNP does not significantly impact on asthma, but may be associated with other lower airway disease [134].

Among the group of nasal polyps, esp. the interleukin(IL)-5 positive endotype, predominantly showing an eosinophilic inflammation, bears a high risk of asthma comorbidity (up to 70%). In these patients, serum total IgE often is increased, independent of the atopic status of the patient [135]. IgE antibodies to Staphylococcus aureus superantigens (SE-IgE) can be detected in a large proportion of these patients, increasing with the severity of disease [102]; about one third to half of the patients suffer from upper airway disease, mostly nasal polyposis. SE-IgE antibodies are significantly associated with severe asthma, oral corticosteroid use, hospitalizations and lung function parameters [102]. SE-IgE antibodies are also associated with an increased risk of suffering from asthma in the general European population according to an epidemiologic study investigating more than 55000 patients [136].

In elderly patients with asthma, diagnostic means therefore should include questions on nasal and sinus symptoms, and a nasal endoscopy and evtl. a CT scan in case of such symptoms. It is advisable to integrate the ENT specialist in the management, once CRS cannot be excluded by the complete lack of nasal symptoms. Tests in serum may include blood eosinophils, total IgE and specific IgE abs to SEs also in SPT-negative subjects. The treatment of the upper airways in these patients might furthermore support the management of the lower airways, and therefore should be part of the individual therapeutic strategy [134].

There is a growing problem of allergic rhinitis in elderly patients. In an epidemiological study of atopic bronchial asthma (BA), allergic rhinitis, and atopic dermatitis (AD) in an elderly Polish population from 16 sites representative of Polish rural and urban areas, the high prevalence of allergic rhinitis and BA in younger individuals with allergies was comparable with those involving groups of elderly Polish patients [137]. The study used medical examinations, an original questionnaire, skin-prick testing (SPT) with common aeroallergens, and serum-specific IgE assays for diagnosis. Similarly, the prevalence of allergic rhinitis among persons aged between 60 to 70 years in Switzerland was around 13-15% [138].

In a study evaluating asthma control in elderly individuals and analyzing the factors that predict poor control, a retrospective, observational study evaluating 108 elderly individuals with asthma was conducted [139]. Clinical data of two groups based on the scores on the asthma control test (ACT), one with ACT scores ≤19 and the second group with ACT scores >19 were studied. Comorbid conditions were found in more than 80% of the patients. Allergic rhinitis was most common comorbid condition (76.9%). In more than one third of the elderly patients with asthma, the asthma was poorly controlled characterized by significantly lower asthma quality of life scores and higher hospitalization rates.

An appropriate assessment and management of upper airway comorbidities in elderly patients with asthma is essential for better asthma control and a better quality of life of the patients.

### Association between asthma and comorbidities

Several studies documented that numerous comorbidities are frequently associated with asthma. Therefore, the identification of comorbidities must become an integral part of the core management of asthma. A systematic evaluation, not only of the presence of comorbid conditions, is necessary, but we have to ensure that these are also adequately treated/controlled so that their effect on asthma is minimized [140].

The AIE was associated with cardiovascular and hypertensive diseases. Also weakly associated with depression, diabetes mellitus, dyslipidemia, osteoporosis and rhino sinusitis. In contrast, it was strongly associated with GORD and, particularly, allergic rhinitis.

Being female slightly increased the association of all cardio- vascular diseases, mainly heart failure, but not angina, coronary disease and acute or old myocardial infarction, with asthma. In males, there was no association between asthma and acute or old myocardial infarction; moreover, in males, asthma was not associated with hypertensive disease. However, in contrast to females, males presented with an association between asthma and angina and coronary disease. In females, the association between diabetes mellitus, dyslipidemia, osteoporosis, depression, psychiatric disorders and GORD and asthma was stronger than in males. In males there was no association between asthma and diabetes whereas the association between asthma and allergic rhinitis and rhino sinusitis was stronger than in females [141].

## Management of asthma: pharmacological and non-pharmacological interventions; asthma education; and pulmonary rehabilitation

Basically, asthma management in the elderly should follow the same rules as for younger patients. The main goals are to achieve asthma control and prevent exacerbations [138]. In these patients, asthma is under-diagnosed and is often confounded with other conditions such as COPD [139]. The evaluation of asthma control/severity may be more difficult. In addition, patients with AIE may be more sensitive to the side-effects of medications [142,143]. As co-morbid conditions are common in this population, polypharmacy is frequent, thereby increasing the risk of drug interactions. Treatment is often suboptimal, due to underassessment of asthma control/severity by the clinician. Multiple patient factors lead to suboptimal disease control, including misunderstanding of asthma as a disease and the treatment regimen, poor adherence to treatment recommendations, memory problems, and socioeconomic challenges [142-145]. Regrettably, most asthma RCTs conducted to date have excluded old adults and the elderly, so no evidence on efficacy and safety of respiratory drugs is available. Therefore, most data come from observational studies.

Acute exacerbation rate in the elderly appears to be comparable to younger adult asthma, as was reported as 21.6% in recent elderly asthma cohort studies [146]. However, factors related to exacerbations may be more multifactorial in the elderly, as they have more comorbidity and decreased socioeconomic, cognitive, or physical capabilities [147]. Therefore, the management of elderly asthma should include further cares for depression, treatment adherence, or inhaler technique education [146].

### Non-pharmacological interventions

Although atopy is less frequent in the elderly, it can still affect a significant number of patients and environmental measures should be considered whenever there is relevant exposure to sensitizers [23,24]. In patients who remain employed, workplace exposures to irritants or sensitizing agents should be documented. The current recommendations on avoidance of respiratory irritants, particularly cigarette smoke, also apply [138].

Aging is associated with weight gain and a sedentary lifestyle. Regular exercise and weight loss in obese asthma patients should be recommended, thereby promoting a healthy lifestyle which is likely to improve the quality of life for the asthmatic [46,148]. Other co-morbid conditions, particularly rhinitis, should be recognized and treated [46,149]. If rhinitis is associated with nasal polyposis and aspirin intolerance, non-steroidal anti-inflammatory agents should be avoided, as they may cause severe bronchoconstriction [150]. Gastroesophageal reflux disease (GERD) should also be considered a potential cause of worsening asthma symptoms although its effects are quite variable within patients [149].

### Pharmacological

Drugs frequently prescribed for cardiovascular conditions such as β-blockers, even in the form of eye drops for conditions such as glaucoma, can induce bronchoconstriction and their use should be reviewed and avoided in asthmatic patients, when these medications can be shown to worsen asthma control [151].

Although there is a lack of studies on the specific effects of current asthma medications in this population, these patients being often excluded from clinical trials, it is nevertheless recommended that treatment should focus on control of airway inflammation as in other asthmatic populations [151-153]. Inhaled corticosteroids (ICS) are the mainstay of asthma treatment and we have no reason to think their efficacy/safety profile should be different in the elderly population. However, there have been reports of underuse of this type of treatment in the elderly [152]. Local side-effects, such as dysphonia and oral candidiasis can be reduced by using a spacer with the metered-dose inhaler and by mouth rinsing after use. Oral corticosteroids use should be minimized to avoid worsening of commonly associated conditions such as osteopenia, diabetes and systemic hypertension [21].

Regarding leukotriene antagonists, although we have few data on their effects in the elderly asthmatic patient, they have an excellent safety profile and can be considered as a second-choice anti-inflammatory drug after ICS, or as add-on therapy [21,154,155]. In this population in particular, each treatment should be considered as a therapeutic trial of size one, and its effects well documented.

The first choice as acute reliever therapy remains an inhaled fast-acting β-adrenergic agonist. It is however even more important in elderly patients to minimize their use as these agents can induce troublesome side-effects such as tremor, tachycardia, or arrhythmias [155]. The sometimes associated reduction in serum potassium and electrocardiographic changes in QT interval may be of concern in cardiac patients, although rarely significant. Long-acting β-adrenergic agonists can be used in association with ICS to improve asthma control in more than mild severity asthmatics, and are usually well tolerated at usual doses although in some patients the doses should be reduced if side-effects are troublesome.

Anticholinergics such as tiotropium are well tolerated in the elderly, but we need more studies about their role in geriatric asthma [156]. They may be considered as add-on therapy to ICS, particularly if long-acting β-adrenergic agonists are not well tolerated. Inhaler technique should be carefully checked and the type of inhaler prescribed may be reassessed if there are some difficulties with its use, for example in cases of severe arthritis, dental problems and general incoordination, or insufficient inspiratory flows.

Nowadays, theophylline are rarely considered in asthma therapy and its use can even be more problematic in the elderly, due to drug interactions and the high potential of side-effects such as arrhythmia.

### Asthma education and treatment adherence

Provision of adequate asthma education is particularly important in the elderly due to the often complex treatments, co-morbidities and sometimes reduced memory and cognitive functions [142,157,158]. Poor treatment adherence, inappropriate inhaler use, or depression were found to be independent predictors for asthma exacerbation in the elderly [158]. An important benefit of education could be in improving adherence to treatment and improving self-management skills. In this regard, adherence to therapy has been often reported as deficient in the elderly. Frequent patient follow-up visits and monitoring for medication adherence, including proper inhalation technique is encouraged. Some patients may also find it difficult to implement a written action plan in the event of an acute exacerbation, and the immediate availability of an asthma educator (by phone or in person), would be ideal [159-161]. The technique of device inhaled medication administration is a difficult problem in elderly patients, and the great majority of elderly patients can not properly use the inhaler, even after the proper instruction [162,163]. The use of dry powder devices, although easy to use, requires the generation of an adequate inspiratory flow can be sub-optimal in fragile patients and those with severe airway obstruction. In such situations, the use of spacers or nebulizer devices may be beneficial. Patients should recognize the rationale behind the use of different drugs, the correct way to use them, and their side effects and polypharmacy with more devices should also be avoided.

### Pulmonary rehabilitation

Pulmonary rehabilitation is mostly recommended for patients with COPD, but some asthmatic patients, particularly when they suffer from the asthma-COPD “overlap syndrome”, may potentially benefit from such program. As exercise is of importance in asthma and COPD, such program may promote active physical activity in this group and previous studies have suggested that this could improve asthma control [164-167].

## Summary and conclusions

There is agreement that AIE is both a common and an under-recognized health problem for the elderly that leads to impairments of lung function and quality of health and life, which is understudied and frequently underdiagnosed and undertreated. There are data to suggest that asthma in older adults is phenotypically different from young patients, with potential impact on the diagnosis, assessment and management in this population. The diagnosis of AIE in older populations relies on the same clinical findings and diagnostic tests used in younger populations, but the interpretation of the clinical data is more challenging. The possibility of multiple causes of symptoms or physical dysfunction is more likely in the aged. The response to drugs may also be less interpretable. There are several reasons why a physician should strive to make a specific diagnosis for a patient presenting with a symptom complex. Just providing the patient with a disease diagnosis or label can be reassuring to the patient. Moreover, advancement of understanding of the epidemiology, natural history, pathobiology, and treatment require a definable disease entity. Whether the threshold for diagnostic criteria is set at a high level of sensitivity, a high level of specificity, or a high level of accuracy depends entirely upon the costs and benefits of an incorrect diagnosis vs. a missed diagnosis.

We need additional information concerning the natural history of physiologic changes in the asthmatic lung with aging, including the development of irreversible airflow obstruction. Furthermore, we need more research to determine if making a clear-cut distinction between asthma vs. COPD in the elderly is important either for prognosis or treatment decisions.

We need better methods, e.g., biomarkers, and tools to help differentiate asthma from other causes of obstructive disease of the airway in the elderly. As physicians start to understand the changes in lung physiology which occur with aging, they will find that it easier to evaluate the older patient who presents with lower airway obstruction.

The challenge today is to encourage new research in AIE but to use the existing knowledge we have to make the diagnosis of AIE, educate the patient, develop a therapeutic approach to control the disease, and ultimately provide a better quality of life to our elderly patients.

## Competing interests

CEBC declares he has no conflicts of interest.

LPB declares he has no conflicts of interest but would like to disclose: He is or has been on the advisory boards of GlaxoSmithKline and Novartis and has received honoraria for speaking from AstraZeneca, GlaxoSmithKline, Merck, and Novartis. Sponsorship for investigator-generated research has come from AstraZeneca, GlaxoSmithKline, Merck Frosst, and Schering; and sponsorship for research funding for participating multicenters has come from Altair, Amgen, Asmacure, AstraZeneca, Boehringer-Ingleheim, Genentech, GlaxoSmithKline, Novartis, Ono Pharma, Pharmaxis, Schering, and Wyeth. He has received support for the production of educational materials from AstraZeneca, GlaxoSmithKline, Merck Frosst, Boehringer-Inglemeim, and Novartis. He has been an advisor for INNESS, the Quebec National Health Institute, and is a member of the Quebec Workmen Compensation Board Respiratory Committee.

PB declares she has no conflicts of interest.

GWC declares he has no competing interests.

JC received a fee for serving as a one-time consultant for Genentech in August 2011 on an issue unrelated to the manuscript, and he receives annual royalties from UpToDate for a book chapter on a topic unrelated to this manuscript.

SHC declares he has no competing interests.

LMF has received consultancy fees from Actelion, Almirall, AstraZeneca, Boehringer Ingelheim, Chiesi Farmaceutici, GlaxoSmithKline, Elevation Pharmaceuticals, Euromediform, Merck Sharp & Dohme, Novartis, Nycomed, OM Pharma,Ferrer Group, Pearl Therapeutics, Roche ,Sigma-Tau Fondazione FADOI – Forest Research Institute. Payment for lectures and support for travel expenses from: AstraZeneca, Boehringer Ingelheim, Chiesi Farmaceutici, Euromediform SrL, GlaxoSmithKline, German Centre for Lung Research, Deutsches Zentrum für Luft und Raumfahrt – German AerospaceCenter, Merck Sharp & Dohme, Menarini, Mundipharma International, Novartis, Nycomed, OM Pharma, Takeda, TEVA Pharmaceuticals, Pfizer, and Sigma-Tau. His institution has received grants from Boehringer Ingelheim, Chiesi Farmaceutici, GlaxoSmithKline, Italian Ministry of Health, Italian Ministry for University and Research, Merck Sharp & Dohme, Nycomed, and Sigma-Tau.

STH declares he has no conflicts of interest.

RK has received compensation from Teva for participation as a speaker and on the advisory board, and from CSL Behring and Novartis for participation on their advisory boards.

DKL has received compensation for speaking from Meda, AstraZeneca, Merck, and Genentech (ongoing). He has received research grants from Teva, Forest, Genentech, Merck, and ViroPharma. He has been compensated for consulting from Shook Hardy Bacon, Saieva and Stine, Genentech, Fowler White Burnett, and Merck. He is on the Advisory Board of Novartis.

AM declares no conflicts of interests.

SP declares he has no conflicts of interests.

KFR has been investigator or co-investigator in projects supported with grants from Altana Pharma, Novartis, AstraZeneca, MSD, and Nycomed. He has received compensation for legal consultation services or expert witness testimony from AstraZeneca, Chiesi Pharmaceutical, Novartis, MSD, and GlaxoSmithKline.

GR has participated as a lecturer, speaker, and advisor in scientific meetings and courses under the sponsorship of Air Products & Chemicals Inc., Almiral, AstraZeneca, Boehringer Ingelheim, Dr. Esteve SA, GlaxoSmithKline, Merck Sharp & Dome, and Novartis.

LJR declares he has no competing interests.

JBS declares she has no competing interests.

AY declares she has no competing interests.

## Authors’ contributions

AY initiated and led the development of the paper as primary author, contributing to all of the sections and unifying the document. SCH and STH were co-project leaders. SHC wrote the Introduction. JC and CEB wrote on the impact of asthma. LPB and GWC wrote on management of asthma. RK, FH, PB, LF, AK, KR, and LR wrote on the aging lung. DKL, SHC, SP, and GJR wrote on diagnosis. JBS wrote on life expectancy. All authors reviewed and approved the final document.
