# Erratum: asthma in the elderly: what we know and what we have yet to know

**DOI:** 10.1186/1939-4551-7-16

**Published:** 2014-06-18

**Authors:** Anahí Yáñez, Sang-Hoen Cho, Joan B Soriano, Lanny J Rosenwasser, Gustavo J Rodrigo, Klaus F Rabe, Stephen Peters, Akio Niimi, Dennis K Ledford, Rohit Katial, Flavia CL Hoyte, Leonardo M Fabbri, Juan C Celedón, Giorgio Walter Canonica, Paula Busse, Louis-Phillippe Boulet, Carlos E Baena-Cagnani, Qutayba Hamid, Claus Bachert, Ruby Pawankar, Stephen T Holgate

**Affiliations:** 1 Investigaciones en Alergia y Enfermedades Respiratorias- InAER, Buenos Aires, Argentina; 2 Department of Internal Medicine, Hanyang University Hospital, Seoul, South Korea; 3 Programa de Epidemiologia e Investigacion Clinica, Fundación Caubet-CIMERA, Illes Balears, Spain; 4 Children’s Mercy Hospital, University of Missouri – Kansas City School of Medicine, Kansas City, Missoui, USA; 5 Departamento de Emergencia, Hospital Central de las Fuerzas Armadas, Montevideo, Uruguay; 6 Krankenhaus Lungen Clinic, Grosshansdorf, Germany; 7 Wake Forest School of Medicine, Winston-Salem, NC, USA; 8 Department of Medical Oncology and Immunology, Nagoya City University Graduate School of Medical Sciences, Kyoto, Japan; 9 Division of Allergy and Immunology, Department of Medicine, Morsani University of South Florida College of Medicine, James A Haley Veterans Hospital, Tampa, FL, USA; 10 Division of Allergy and Immunology, National Jewish Health, Denver, CO, USA; 11 Department of Oncology, Haematology, and Respiratory Diseases, University of Modena and Reggio Emilia, Modena, Italy; 12 Division of Pulmonary Medicine, Allergy and Immunology, Children’s Hospital of UPMC, Pittsburgh, PA, USA; 13 Respiratory Diseases and Allergy, University of Genoa, Genoa, Italy; 14 Division of Clinical Immunology, Department of Medicine, Mount Sinai School of Medicine, New York, NY, USA; 15 Institut universitaire de cardiologie et de pneumologie de Québec, Quebec Heart and Lung Institute, Laval University, Quebéc, Canada; 16 Centre for Research in Respiratory Medicine, Catholic University of Córdoba, Córdoba, Argentina; 17 Meakins-Christie Laboratories, McGill University, Quebéc, Canada; 18 Upper Airways Research Laboratory (URL), Clinics ENT-Department, University Hospital Ghent, Ghent, Belgium; 19 Department of Pediatrics, Nippon Medical School, Tokyo, Japan; 20 Faculty of Medicine Clinical and Experimental Sciences, University of Southampton, Hampshire, United Kingdom

## Erratum

Following the publication of our article [1], we noticed that Dr Flavia C. L. Hoyte had inadvertently been omitted from the author list. FCLH declares she has no conflicts of interest. The author list has now been corrected and the amended authors contributions section included below.

AY initiated and led the development of the paper as primary author, contributing to all of the sections and unifying the document. SCH and STH were co-project leaders. SHC wrote the Introduction. JC and CEB wrote on the impact of asthma. LPB and GWC wrote on management of asthma. RK, FCLH, PB, LF, AK, KR, and LR wrote on the aging lung. DKL, SHC, SP, and GJR wrote on diagnosis. JBS wrote on life expectancy. All authors reviewed and approved the final document.
